# Physical Activity Levels of a Multi-Ethnic Population of Young Men Living in Saudi Arabia and Factors Associated With Physical Inactivity

**DOI:** 10.3389/fpubh.2021.734968

**Published:** 2022-02-02

**Authors:** Jozaa Z. AlTamimi, Reham I. Alagal, Nora M. AlKehayez, Naseem M. Alshwaiyat, Hamid A. Al-Jamal, Nora A. AlFaris

**Affiliations:** ^1^Department of Physical Sports Sciences, College of Education, Princess Nourah Bint Abdulrahman University, Riyadh, Saudi Arabia; ^2^School of Nutrition and Dietetics, Faculty of Health Sciences, Universiti Sultan Zainal Abidin, Terengganu, Malaysia; ^3^School of Biomedicine, Faculty of Health Sciences, Universiti Sultan Zainal Abidin, Terengganu, Malaysia

**Keywords:** physical activity, physical inactivity, body mass index, young men, multi-ethnic, Saudi Arabia

## Abstract

**Objective:**

Regular physical activity is essential for lifelong optimal health. Contrarily, physical inactivity is linked with risk for many chronic diseases. This study was conducted to evaluate the physical activity levels and factors associated with physical inactivity among a multi-ethnic population of young men living in Saudi Arabia.

**Methods:**

This is a cross-sectional study involving 3,600 young men (20–35 years) living in Riyadh, Saudi Arabia. Sociodemographic and physical activity data were collected from subjects by face-to-face interviews. Physical activity characteristics were evaluated by using the Global Physical Activity Questionnaire. Weight and height were measured following standardized methods, then body mass index was calculated.

**Results:**

Physical inactivity was reported among 24.9% of study subjects. The lowest and highest rates of physical inactivity were reported among subjects from the Philippines (14.0%) and Saudi Arabia (41.5%), respectively. There is a high variation in daily minutes spent on physical activities related to work, transport, recreation, vigorous and moderate-intensity physical activities and sedentary behaviors among study participants based on their nationalities. Nationality, increasing age, longer residency period in Saudi Arabia, living within a family household, having a high education level, earning a high monthly income, and increasing body mass index were significantly associated with a higher risk of physical inactivity among the study participants.

**Conclusion:**

Physical inactivity prevalence is relatively high among a multi-ethnic population of young men living in Saudi Arabia. The findings confirmed notable disparities in the physical activity characteristics among participants from different countries living in Saudi Arabia.

## Introduction

Physical activity is defined as any voluntary body movement performed by skeletal muscles and needs energy expenditure higher than the basal level (1). Regular physical activity has long been recognized as a protective factor in preventing and treating the most common chronic diseases, such as obesity, cardiovascular disease, type 2 diabetes, and breast and colon cancers (2). It also plays a vital function in improving mental health and overall quality of life (3, 4). On the other hand, the lack of moderate to high physical activity in a person's lifestyle is defined as physical inactivity (5). Current evidence emphasized that over one-fourth of the world's adults are physically inactive (6). Physical inactivity has been recognized as a global public health problem and linked to increased morbidity and overall mortality among adults all over the world (7, 8). This has prompted the World Health Organization (WHO) to target a 15% decrease in the global prevalence of physical inactivity in adults and adolescents by 2030 (5).

Saudi Arabia has experienced a rapid socio-economic transition in recent decades, which has coincided with changes in Saudi people's lifestyle toward sedentary behaviors due to urbanization and motorization (9). These lifestyle changes are occurring simultaneously with the rise in prevalence of obesity and other chronic diseases among Saudi people (10). In fact, the rise in obesity and chronic diseases in Saudi Arabia is primarily due to increased intake of unhealthy foods and a decrease in physical activity levels (9, 11, 12).

Interestingly, physical inactivity rates among adults vary greatly within and between countries, with certain adult subpopulations reporting rates as high as 80%. The Eastern Mediterranean, the Americas, Europe, and the Western Pacific areas have the highest rates of physical inactivity among adults, whereas the South-East Asia region has the lowest (13). These rates could be influenced by several factors such as economic growth, transportation patterns, technology use, and accepted cultural standards. During the previous few decades, Saudi Arabia has seen a tremendous influx of migrants, most of whom were young men from various Middle Eastern and Asian countries (14). Existing literature does not provide adequate information about physical activity levels and factors associated with physical inactivity among the multi-ethnic population of young men living in Saudi Arabia. Therefore, the aim of the current study was to evaluate physical activity levels and factors associated with physical inactivity among young men from Saudi Arabia and eleven Middle Eastern and Asian countries.

## Methods

### Study Design and Subjects

This study is part of a larger research project entitled as the Relationship between Obesity, physical Activity, and Dietary pattern among men in Saudi Arabia (ROAD-KSA). It is a cross-sectional study that aimed to determine the prevalence of overweight and obesity, physical activity levels, and dietary patterns among young and middle-aged men from Saudi Arabia (reference group) and 11 Middle Eastern and Asian countries (Egypt, Yemen, Syria, Jordan, Sudan, Turkey, Pakistan, Afghanistan, India, Bangladesh, and the Philippines) residing in Riyadh, Saudi Arabia, as well as the correlations between these factors. The current research was conducted in Riyadh, Saudi Arabia.

Study subjects were chosen randomly from public sites in Riyadh, including public gardens and shopping centers, using a stratified clustered sampling technique based on geographic areas in Riyadh. The Riyadh city was stratified into five areas (east, west, north, south and center), and each area was clustered into several neighborhoods. Two neighborhoods from each area were chosen randomly. Public gardens and shopping centers in the selected neighborhoods were targeted to recruit our participants randomly. The eligibility for participation includes young men between the ages of 20 and 35 who live in Riyadh, are free of any physical impairment, and have a single nationality from one of the following countries (Saudi Arabia, Egypt, Yemen, Syria, Jordan, Sudan, Turkey, Pakistan, Afghanistan, India, Bangladesh, and the Philippines). Former to recruitment, subjects signed an informed consent form as required by the Helsinki Declaration. The research ethics committee of Princess Nourah bint Abdulrahman University in Riyadh, Saudi Arabia, approved the study procedure.

### Sociodemographic Parameters

Face-to-face interviews were used to collect sociodemographic data from study participants by trained researchers. Participants' nationality, age, length of stay in Saudi Arabia, household type, marital status, educational level, and monthly income are the sociodemographic parameters collected.

### Body Mass Index

The weight and height of study subjects were measured by trained researchers. A training session about standardized methods for weight and height measurement was organized for all research team members before data collection to minimize personnel measurement bias. A calibrated digital weight scale was used to measure the body weight to the closest 100 g while wearing little clothing and without shoes. Furthermore, the subject's body height was measured to the closest 1 mm in full standing posture without shoes using a calibrated portable stadiometer. Body mass index (BMI) was computed by dividing weight (kg) by height (m^2^) (15).

### Physical Activity Evaluation

Physical activity levels of subjects were evaluated by using the Global Physical Activity Questionnaire (GPAQ, version 2.0) developed by WHO (16). It has acceptable reliability and validity in measuring the physical activity of adults in surveillance studies (17). The GPAQ consists of 16 questions and collects information on physical activity participation in three different domains (work, transport, and recreation) and sedentary behavior. The intensity of physical activities studied with GPAQ is divided into two categories: moderate and vigorous-intensity activities. Physical activities that involve hard physical effort and create significant increases in respiration or heart rate are classified as vigorous-intensity activities, while physical activities that involve moderate physical effort and induce slight increases in breathing or heart rate are classified as moderate-intensity (18). The Metabolic Equivalent of Tasks (METs) is a standard unit of measurement for expressing physical activity intensity. When using GPAQ data to calculate a person's overall energy expenditure, time spent in moderate-intensity physical activities is given four METs, and time spent in vigorous-intensity physical activities is given eight METs. The GPAQ's first and third domains inquired about the number of usual weekly days and typical daily periods spent on vigorous and moderate-intensity work and recreation activities, respectively. The GPAQ's second domain inquired about the number of average weekly days and typical daily times spent on moderate-intensity transportation activities (18). The GPAQ also asked about typical daily times spent on sedentary behaviors. Sedentary behaviors are defined as sitting or reclining at work or home, including time spent travelling by vehicles, reading or watching television, but do not include time spent sleeping (18). The physical activity data were collected by face-to-face interviews conducted by trained researchers. Showcards developed by WHO for each type of activity covered by the GPAQ were used during physical activity data collection to help subjects recognize the domain of physical activity meant by each question.

According to the GPAQ analysis guide, The GPAQ categorized physical activity into three levels (high, moderate and low) based on specific criteria. The physical activity level is classified as high if a person reported vigorous-intensity activity on at least 3 days, with a minimum of 1,500 MET-minutes per week or seven or more days of any combination of walking or moderate or vigorous-intensity activities, with a minimum of 3,000 MET-minutes per week. The level of physical activity is classified as moderate if a person reported three or more days of vigorous-intensity activity of at least 20 min per day or 5 or more days of moderate-intensity activity of at least 30 min per day or 5 or more days of any combination of walking, moderate or vigorous-intensity activities achieving a minimum of 600 MET-minutes per week. Otherwise, the level of physical activity is classified as low if the above criteria were not satisfied (18). Subjects with low physical activity were considered physically inactive, while subjects with moderate or high physical activity were considered physically active (5, 19). Furthermore, the GPAQ data analysis results examined if WHO recommendations for physical activity had been met or not by each participant. WHO recommendations on physical activity for health include doing at least 150 min per week of moderate-intensity physical activity or 75 min per week of vigorous-intensity physical activity or equivalent combination of moderate- and vigorous-intensity physical activity achieving at least 600 MET-minutes per week (18). Other results generated from the GPAQ data analysis include total daily minutes spent on physical activity, daily minutes spent on various physical activity domains (work, transportation and recreation), daily minutes spent on vigorous and moderate-intensity physical activities, the proportion of daily minutes spent on various physical activity domains from total daily minutes spent on physical activity, the proportion of daily minutes spent on vigorous and moderate-intensity physical activities from total daily minutes spent on physical activity, percent of participants doing no physical activities related to various physical activity domains, and percent of participants doing no physical activities related to vigorous and moderate-intensity physical activities (18).

### Statistical Analysis

IBM SPSS Statistics for Windows (version 26. Armonk, New York, United States, 2019) was used for data analysis. The method of cleaning and scoring GPAQ data has been described in detail elsewhere (18). There is no missing data. Statistical analysis for physical activity parameters was carried out for the complete study sample of subjects and the study sample subgroups after stratifying study participants based on their nationality. Categorical variables were analyzed by using the Chi-squared test and presented as numbers and percentages. Continuous variables were analyzed by using the one-way ANOVA test and presented as means and standard deviations. The Tukey *post hoc* test was used to determine significant differences between subjects with different nationalities. Univariate and multivariate logistic regression analyses were performed to detect the factors related to physical inactivity risk. All reported *P-*values were made based on two-tailed tests. Differences were considered statistically significant at *P* < 0.05.

## Results

The sociodemographic characteristics and BMI of the study subjects are presented in Table 1. The current study included 3600 young men from Saudi Arabia and 11 Middle Eastern and Asian countries living in Riyadh, Saudi Arabia. Physical activity characteristics of study subjects and subjects stratified by nationalities are presented in Table 2. Low physical activity level (physical inactivity) was reported among 24.9% of study subjects. By nationalities, the lowest and highest rates of low physical activity level were reported among subjects from the Philippines (14.0%) and Saudi Arabia (41.5%), respectively. Furthermore, moderate and high physical activity levels were reported among 26.7 and 48.4% of study subjects, respectively. A relatively high rate of study participants (83.0%) was found meeting WHO recommendations on physical activity for health.

**Table 1:** Sociodemographic characteristics and body mass index of all study subjects (*n* = 3,600).

**Variables**	**N / Mean**	**% / SD**
Participants nationality
Saudi (reference group)	289	8.0%
Egyptian	289	8.0%
Yemeni	335	9.3%
Syrian	293	8.1%
Jordanian	280	7.8%
Sudanese	276	7.7%
Turkish	203	5.6%
Pakistani	306	8.5%
Afghan	303	8.4%
Indian	297	8.3%
Bangladeshi	350	9.7%
Filipino	379	10.5%
Age (years)	29.6	3.2
Residency Period in Saudi Arabia (years)	7.2	7.0
Household type
Non-family household	2,920	81.1%
Family household	680	18.9%
Marital status
Single	1,919	53.3%
Married	1,681	46.7%
Education level
High school or less	2,284	63.4%
College degree or more	1,316	36.6%
Monthly income
Low (<1000 USD)	2,630	73.1%
High (≥1000 USD)	970	26.9%
Body Mass Index (kg/m^2^)	25.1	3.2

**Table 2:** Physical activity characteristics of all study subjects (*n* = 3,600) and subjects stratified by nationalities.

Variables^*^	**Total *n* = 3,600**	**Saudi Arabia *n* = 289**	**Egypt *n* = 289**	**Yemen *n* = 335**	**Syria *n* = 293**	**Jordan *n* = 280**	**Sudan *n* = 276**
Physical activity levels							
Low	896 (24.9%)	120 (41.5%)	60 (20.8%)	97 (29.0%)	78 (26.6%)	60 (21.4%)	41 (14.9%)
Moderate	962 (26.7%)	81 (28.0%)	67 (23.2%)	63 (18.8%)	67 (22.9%)	146 (52.1%)	18 (6.5%)
High	1,742 (48.4%)	88 (30.4%)	162 (56.1%)	175 (52.2%)	148 (50.5%)	74 (26.4%)	217 (78.6%)
Respondents meeting WHO recommendations^**^	2,987 (83.0%)	214 (74.0%)	250 (86.5%)	256 (76.4%)	242 (82.6%)	242 (86.4%)	252 (91.3%)
Daily minutes spent on activities related to							
Work	128.8 (180.8)	41.5 (87.0)ab	166.5 (197.7)e	141.3 (194.6)de	99.5 (148.2)cd	20.1 (40.3)a	230.1 (171.4)f
Transport	32.1 (64.1)	10.3 (20.1)a	15.8 (25.5)ab	21.0 (53.8)ab	16.7 (36.1)ab	19.1 (21.4)ab	14.6 (13.0)ab
Recreation	14.3 (25.5)	28.9 (44.8)f	11.1 (22.7)bcd	14.6 (30.5)d	22.5 (30.2)ef	16.1 (23.5)de	4.2 (11.2)a
Total	175.2 (209.8)	80.8 (111.1)ab	193.5 (195.8)e	176.9 (203.0)de	138.8 (158.3)cd	55.2 (56.3)a	248.8 (171.3)f
Vigorous activities	88.1 (162.1)	21.0 (43.4)a	104.5 (158.1)cd	66.9 (133.3)bc	42.5 (77.9)ab	15.8 (30.8)a	148.1 (182.7)e
Moderate activities	87.0 (113.3)	59.8 (89.0)abc	89.0 (126.5)cde	110.0 (147.8)e	96.3 (124.8)de	39.4 (47.3)a	100.7 (143.4)de
Sedentary behaviors	260.1 (162.9)	413.5 (184.3)f	422.3 (171.8)f	330.6 (153.9)e	347.2 (191.5)e	286.7 (116.5)d	140.9 (89.6)a
Proportion of minutes spent in physical activities related to							
Work	0.51 (0.39)	0.33 (0.37)ab	0.61 (0.40)de	0.58 (0.39)cde	0.49 (0.41)c	0.27 (0.30)a	0.81 (0.29)f
Transport	0.29 (0.33)	0.23 (0.32)bc	0.23 (0.32)c	0.24 (0.33)c	0.21 (0.30)bc	0.41 (0.33)d	0.12 (0.23)a
Recreation	0.20 (0.30)	0.45 (0.37)f	0.16 (0.28)bc	0.18 (0.30)bcd	0.31 (0.35)e	0.32 (0.31)e	0.07 (0.20)a
Vigorous activities	0.32 (0.38)	0.26 (0.34)abc	0.38 (0.40)d	0.30 (0.36)cd	0.30 (0.33)cd	0.23 (0.31)abc	0.52 (0.46)e
Moderate activities	0.68 (0.38)	0.75 (0.34)cde	0.62 (0.40)b	0.70 (0.36)bc	0.70 (0.33)bc	0.77 (0.31)cde	0.48 (0.46)a
Percentage of participants doing no physical activities related to							
Work	1,204 (33.4%)	152 (52.6%)	81 (28.0%)	117 (34.9%)	114 (38.9%)	126 (45.0%)	28 (10.1%)
Transport	1,192 (33.1%)	156 (54.0%)	97 (33.6%)	139 (41.5%)	134 (45.7%)	74 (26.4%)	62 (22.5%)
Recreation	1,674 (46.5%)	85 (29.4%)	150 (51.9%)	169 (50.4%)	104 (35.5%)	82 (29.3%)	229 (83.0%)
Vigorous activities	1,810 (50.3%)	163 (56.4%)	115 (39.8%)	154 (46.0%)	122 (41.6%)	151 (53.9%)	120 (43.5%)
Moderate activities	457 (12.7%)	57 (19.7%)	30 (10.4%)	61 (18.2%)	49 (16.7%)	13 (4.6%)	25 (9.1%)
Variables^*^	**Turkey** ***n*** **=** **203**	**Pakistan** ***n*** **=** **306**	**Afghanistan** ***n*** **=** **303**	**India** ***n*** **=** **297**	**Bangladesh** ***n*** **=** **350**	**Philippines** ***n*** **=** **379**	***P*** **value**
Physical activity levels							
Low	69 (34.0%)	70 (22.9%)	67 (22.1%)	103 (34.7%)	78 (22.3%)	53 (14.0%)	0.001
Moderate	65 (32.0%)	42 (13.7%)	28 (9.2%)	88 (29.6%)	108 (30.9%)	189 (49.9%)	
High	69 (34.0%)	194 (63.4%)	208 (68.6%)	106 (35.7%)	164 (46.9%)	137 (36.1%)	
Respondents meeting WHO recommendations^**^	149 (73.4%)	254 (83.0%)	256 (84.5%)	208 (70.0%)	314 (89.7%)	350 (92.3%)	0.001
Daily minutes spent on activities related to							
Work	78.5 (105.2)bc	263.1 (242.5)f	257.0 (230.3)f	58.6 (81.6)abc	156.5 (187.6)e	30.2 (42.4)a	0.001
Transport	20.4 (44.4)ab	37.7 (50.0)cd	40.3 (47.4)d	23.8 (50.2)abc	117.9 (141.5)e	27.4 (25.3)bcd	0.001
Recreation	7.9 (21.8)abc	21.9 (24.7)e	16.4 (19.7)de	6.4 (14.6)ab	6.0 (13.0)ab	14.1 (19.0)cd	0.001
Total	106.8 (120.8)bc	322.8 (285.8)g	313.8 (261.7)g	88.9 (102.0)abc	280.6 (288.1)fg	71.6 (53.5)ab	0.001
Vigorous activities	29.6 (78.0)ab	225.5 (251.9)f	219.6 (225.5)f	25.1 (61.9)a	132.8 (185.2)de	13.9 (30.2)a	0.001
Moderate activities	77.2 (95.2)bcd	97.3 (92.7)de	94.2 (96.3)de	63.7 (89.9)abc	147.6 (147.8)f	57.8 (50.3)ab	0.001
Sedentary behaviors	141.6 (93.1)a	251.4 (95.6)bc	276.8 (131.6)cd	132.0 (74.0)a	139.1 (68.2)a	225.9 (99.0)b	0.001
Proportion of minutes spent in physical activities related to							
Work	0.55 (0.40)cd	0.67 (0.35)e	0.66 (0.36)e	0.54 (0.35)cd	0.37 (0.37)b	0.34 (0.30)ab	0.001
Transport	0.33 (0.36)d	0.14 (0.23)ab	0.17 (0.22)abc	0.36 (0.31)d	0.52 (0.39)e	0.42 (0.30)d	0.001
Recreation	0.11 (0.24)abc	0.19 (0.30)cd	0.17 (0.30)bcd	0.10 (0.22)ab	0.11 (0.25)ab	0.24 (0.26)de	0.001
Vigorous activities	0.19 (0.34)ab	0.51 (0.39)e	0.54 (0.37)e	0.19 (0.34)ab	0.29 (0.36)bcd	0.18 (0.27)a	0.001
Moderate activities	0.81 (0.34)de	0.49 (0.39)a	0.46 (0.37)a	0.81 (0.34)de	0.71 (0.36)bcd	0.82 (0.27)e	0.001
Percentage of participants doing no physical activities related to							
Work	75 (36.9%)	88 (28.8%)	75 (24.8%)	85 (28.6%)	153 (43.7%)	110 (29.0%)	0.001
Transport	76 (37.4%)	158 (51.6%)	108 (35.6%)	78 (26.3%)	49 (14.0%)	61 (16.1%)	0.001
Recreation	134 (66.0%)	89 (29.1%)	82 (27.1%)	195 (65.7%)	261 (74.6%)	94 (24.8%)	0.001
Vigorous activities	145 (71.4%)	116 (37.9%)	92 (30.4%)	215 (72.4%)	198 (56.6%)	219 (57.8%)	0.001
Moderate activities	38 (18.7%)	71 (23.2%)	45 (14.9%)	49 (16.5%)	13 (3.7%)	6 (1.6%)	0.001

* *Categorical variables were analyzed by using Chi-squared test and expressed as numbers and percentages. Continuous variables were analyzed by using one-way ANOVA test and expressed as means and standard deviations. Tukey post hoc test was used to determine significant differences between sub-groups. Means that were subscripted with different letters are significantly different at P < 0.05*.

** *WHO recommendations on physical activity for health is at least 150 min per week of moderate-intensity physical activity or 75 min per week of vigorous-intensity physical activity or equivalent (at least 600 MET–minutes per week; MET means the metabolic equivalent of task)*.

The mean total daily minutes spent on physical activity by study subjects was 175.2 ± 209.8 min, whilst the mean daily minutes spent on physical activities related to work, transport, recreation, vigorous-intensity physical activities and moderate-intensity physical activities were 128.8 ± 180.8, 32.1 ± 64.1, 14.3 ± 25.5, 88.1 ± 162.1, and 87.0 ± 113.3 min, respectively. High variation was observed among participants from different countries regard daily minutes spent on physical activities related to work (20.1 min for Jordanian subjects and 263.1 min for Pakistani subjects), transport (10.3 min for Saudi subjects and 117.9 min for Bangladeshi subjects), recreation (4.2 min for Sudanese subjects and 28.9 min for Saudi subjects), vigorous-intensity physical activities (13.9 min for Filipino subjects and 225.5 min for Pakistani subjects), moderate-intensity physical activities (39.4 min for Jordanian subjects and 147.6 min for Bangladeshi subjects), and sedentary behaviors (132.0 min for Indian subjects and 422.3 min for Egyptian subjects).

The means of the proportion of weekly minutes spent in physical activities related to work, transport, and recreation from total physical activity were 0.51 ± 0.39, 0.29 ± 0.33, and 0.20 ± 0.30, respectively. Similarly, the means of the proportion of weekly minutes spent in vigorous-intensity physical activities and moderate-intensity physical activities from total physical activity were 0.32 ± 0.38 and 0.68 ± 0.38, respectively. Moreover, the percentages of participants who did not engage in any physical activity related to work, transport, recreation, vigorous-intensity physical activities, and moderate-intensity physical activities were 33.4, 33.1, 46.5, 50.3, and 12.7%, respectively.

The risk of physical inactivity among all subjects for sociodemographic characteristics and BMI is shown in Table 3. Compared with subjects from the Philippines, subject form several other countries had a significantly higher risk of being physically inactive, including Saudi Arabia [adjusted odds ratio (OR) = 10.50, *P* = 0.001], Egypt (unadjusted OR = 1.61, *P* = 0.021), Yemen (adjusted OR = 3.09, *P* = 0.001), Syria (adjusted OR = 1.93, *P* = 0.003), Jordan (unadjusted OR = 1.68, *P* = 0.013), Turkey (adjusted OR = 5.08, *P* = 0.001), Pakistan (adjusted OR = 2.45, *P* = 0.001), Afghanistan (adjusted OR = 2.40, *P* = 0.001), India (adjusted OR = 5.19, *P* = 0.001), and Bangladesh (adjusted OR = 2.77, *P* = 0.001). Moreover, increasing age was significantly associated with a higher risk of physical inactivity (adjusted OR = 1.05, *P* = 0.002). Similarly, longer residency period in Saudi Arabia was significantly associated with a higher risk of physical inactivity (adjusted OR = 1.02, *P* = 0.001). Subjects those who live within a family household had a significantly higher risk of physical inactivity compared with those who live within non-family household (adjusted OR = 1.46, *P* = 0.001). Participants have at least a college degree had a significantly higher risk of physical inactivity compared with those with lower education level (adjusted OR = 1.59, *P* = 0.001). In the same fashion, participants having high monthly income (1,000 USD or more) had a significantly higher risk of physical inactivity compared with those who have low monthly income (unadjusted OR = 1.47, *P* = 0.001). Finally, Increasing BMI was significantly associated with a higher risk of physical inactivity (adjusted OR = 1.04, *P* = 0.002).

**Table 3:** Risk of physical inactivity among all subjects (*n* = 3,600) for sociodemographic characteristics and body mass index.

**Variables**	Unadjusted odds ratio^*^	**95% CI**	***P*-value**	Adjusted odds ratio^**^	**95% CI**	***P*-value**
Participants nationality
Filipino	1.00			1.00		
Saudi	4.37	3.01–6.34	**0.001**	10.50	4.48–24.63	**0.001**
Egyptian	1.61	1.07–2.42	**0.021**	1.40	0.91–2.14	0.123
Yemeni	2.51	1.72–3.65	**0.001**	3.09	2.03–4.69	**0.001**
Syrian	2.23	1.51–3.29	**0.001**	1.93	1.25–2.98	**0.003**
Jordanian	1.68	1.12–2.52	**0.013**	1.40	0.90–2.18	0.135
Sudanese	1.07	0.69–1.67	0.754	1.34	0.83–2.16	0.227
Turkish	3.17	2.10–4.78	**0.001**	5.08	3.13–8.23	**0.001**
Pakistani	1.82	1.23–2.71	**0.003**	2.45	1.58–2.82	**0.001**
Afghan	1.75	1.17–2.60	**0.006**	2.40	1.53–3.78	**0.001**
Indian	3.27	2.24–4.76	**0.001**	5.19	3.37–8.01	**0.001**
Bangladeshi	1.76	1.20–2.59	**0.004**	2.77	1.79–4.31	**0.001**
Age (years)	1.03	1.00–1.05	**0.038**	1.05	1.02–1.08	**0.002**
Residency period in Saudi Arabia (years)	1.03	1.02–1.04	**0.001**	1.02	1.01–1.03	**0.001**
Household type
Non-family household	1.00			1.00		
Family household	1.69	1.41–2.02	**0.001**	1.46	1.12–1.90	**0.005**
Marital status
Single	1.00			1.00		
Married	0.99	0.85–1.15	0.854	0.83	0.69–1.01	0.057
Education level
High school or less	1.00			1.00		
College degree or more	1.21	1.04–1.42	**0.015**	1.59	1.24–2.05	**0.001**
Monthly income
Low (<1,000 USD)	1.00			1.00		
High (≥1,000 USD)	1.47	1.24–1.73	**0.001**	1.13	0.92–1.39	0.247
Body mass index (kg/m^2^)	1.04	1.01–1.06	**0.001**	1.04	1.01–1.06	**0.002**

* *Univariate logistic regression analysis was used to test differences between physically inactive subjects vs. physically active subjects (reference group). Differences were considered statistically significant at P < 0.05, and significant values were presented in Bold type*.

** *Multivariate logistic regression analysis was used to test differences between physically inactive subjects vs. physically active subjects (reference group) after adjusting for subjects' sociodemographic characteristics and body mass index. Differences were considered statistically significant at P < 0.05, and significant values were presented in Bold type*.

## Discussion

This study provides a clear overview of physical activity levels among a multi-ethnic population of young men from Saudi Arabia and 11 Middle Eastern and Asian countries living in Saudi Arabia. According to the findings, around one-fourth of young men living in Saudi Arabia are physically inactive. Saudi Arabia has high physical inactivity prevalence rates at the global level (9). Regular physical activity is a challenging choice for Saudis due to several barriers, including lack of motivation, increased urbanization, heavy traffic, hot arid weather, cultural hurdles, lack of social support, and limited time and resources (9). Hence, the Saudi population's daily physical activity has decreased, leading to an increasing prevalence of physical inactivity, and consequently, the risk of chronic diseases (10). Physical inactivity was reported among 66.6% of the overall population in Saudi Arabia (60.1% for men and 72.9% for women), while only 16.8% and 16.6%of the population engaged in moderate and high levels of physical activity, respectively (19). A large population-based study conducted in Saudi Arabia found that the prevalence of physical inactivity among Saudi adults was 96.1% (93.9% among men and 98.1% among women) (20). Another study reported that 56.3% of male college students aged 17 to 25 years were physically inactive (21). A recent population-based study carried out also in Saudi Arabia reported that the rates of physical inactivity were 82.6% among Saudis aged 15 years or more (71.7% of Saudi males and 91.1% of Saudi females) and 86.1% of non-Saudi counterpart residents (83.9% of non-Saudi males and 92.0% of non-Saudi females) (22).

Saudi Arabia is one of the Middle East's fastest-growing high-income countries. Consequently, Saudi Arabia attracts workers from all over the world, mainly from the Middle East, South Asia, and Southeast Asia. In 2013, expatriates made up 56.5% of the total workforce and 89% of the private sector employment in Saudi Arabia (14). Non-Saudi residents make up roughly 31% of the population of Saudi Arabia. Furthermore, males accounted for almost 70% of the non-Saudi population in Saudi Arabia (23). This is especially fascinating because the presence of migrants from diverse countries allows for a cross-sectional study of variations between their populations in various areas, such as lifestyle patterns and health markers.

The current study results revealed significant disparities in physical activity parameters among young men living in Saudi Arabia from various countries. Certain lifestyle characteristics, such as work type, transportation, leisure time activities, and the intensity and duration of physical activity, could explain these discrepancies (12, 24). Manual labor jobs such as working in construction, medical emergency, and firefighting are typically connected with higher physical activity levels than office labor jobs such as working in the education sector, banking and call answering services (25). For example, most Saudi young men work in office labor jobs. In contrast, most Pakistani young men living in Saudi Arabia work in manual labor jobs. Our results confirmed this observation as there is a notable difference in work-related physical activity among Saudi and Pakistani young men. Furthermore, common transportation ways can influence people's physical activity levels. Using automated vehicles for short distance travels, walking or riding a bicycle has been related to higher physical activity levels (26). In fact, most Bangladeshi young men living in Saudi Arabia commute short travels by bicycle. Contrarily, Saudi young men rely heavily on their automobiles for transportation, even short trips. Current study findings supported this observation as there is a variation in transport-related physical activity among Saudi and Bangladeshi young men. Cultural values, available free time, and available resources and relevant sites for completing workouts and engaging in recreational physical activities influence the leisure time physical activities of young men from various countries (27, 28). For example, young Saudi men were more engaged in recreational physical activities than young Sudanese men living in Saudi Arabia. In fact, examining these disparities in physical activity characteristics provides a chance to identify and implement appropriate measures to minimize physical inactivity in high-prevalence groups.

The monitoring of physical activity levels and associated risk factors for different community groups is an important aspect of health-promoting initiatives to combat physical inactivity (29). Our results revealed that several sociodemographic factors were significantly associated with a higher risk of physical inactivity. The nationality of subjects was one of these factors, and this could be refed to cross-cultural variation among young men from different countries in occupations, common transportation ways and lifestyles including typical leisure time activities (13). Increasing age was also found to play a crucial role in physical inactivity. Young adults with ageing usually tend to do less physical activity and have more sedentary behaviors (30). This is consistent with previously reported results in Saudi Arabia (20, 22, 31). A longer residency period in Saudi Arabia was significantly associated with a higher risk of physical inactivity, and this could be explained by urbanization and motorization commonly seen in Saudi Arabia and their effect on people lifestyles (9, 12). This result was in line with previous studies from Saudi Arabia (32, 33). The health of emigrants is thought to decline with the length of time they spend in a new host country. Cultural differences, interpersonal and economic changes, and lifestyle changes, including eating habits and physical activity associated with migration, may all contribute to health problems (34). Living within a family household was also significantly associated with a higher risk of physical inactivity. Social gathering is a common feature of family structure in Saudi Arabia. Unfortunately, these gatherings focus mainly on sedentary behaviors such as eating meals and watching television (9). Higher education and higher income were significantly associated with a higher risk of physical inactivity. In Saudi Arabia, educated and/or wealthy men typically have office labor jobs with sitting for long hours and using automobiles for transportation, which may be physically inactive (25). Finally, only marital status was not significantly associated with the risk of physical inactivity. A similar result was reported in a previous study (35).

Saudi Arabia has one of the highest rates of overweight and obesity in the world (10, 36). It is well-known that a lack of physical activity is an important variable that can lead to obesity (37). High overweight and obesity rates were seen in the Saudi population could be accused largely to highly prevalent physical inactivity and sedentary lifestyles (38). Our results revealed that increasing BMI was significantly associated with a higher risk of physical inactivity among the study subjects. This result was consistent with previous studies from Saudi Arabia (20, 25, 31, 39, 40).

Notably, Saudi Arabia's population is considered a young population, with 72% of the population aged 34 or younger (23). In Saudi Arabia, young Saudi men aged 20 to 34 years made up around 30% of the male population, whereas young non-Saudi men aged 20 to 34 years made up around 38% of all non-Saudi male residents (23). Generally, young adults are particularly vulnerable to physical inactivity for several reasons, including time limitations, lack of accessible and suitable sports places, having other important priorities, lack of friends to encourage and lack of motivation (21, 41). Sedentary behaviors became widespread during this stage of life. These sedentary behaviors usually seen among young adults include computer use, television watching, reading, electronic gaming and smartphone use (42, 43). Unfortunately, physical inactivity and sedentary behaviors are connected with higher rates of health problems (7, 8). The significant incidence of physical inactivity among young men should raise alarm bells for health officials, especially when considering that most physically inactive young men maintained sedentary lifestyles (21). Health education for young adults to encourage them to engage in regular physical activities is critical for lifelong optimal health (44). This could be challenging due to a lack of motivation and time to engage in an active lifestyle (28). As a result, developing appropriate intervention programs and making physical activity counseling a priority in clinical practice is crucial (45). In Saudi Arabia, there were several initiatives aimed at encouraging physical activity. The majority of them were disjointed, short-term efforts that lacked a coordinating body and objective appraisals of their outcomes. There is a need for a national strategy that encourages active living while discouraging sedentary behavior, with input from all parties concerned (46).

There are certain limitations to this study that should be considered. One of the drawbacks of a cross-sectional design is that it cannot determine causality. Another limitation is that the core variables, physical activity parameters, are self-reported outcome measures prone to recall and social desirability biases. To effectively quantify the prevalence of physical activity, objective methods such as accelerometers are required. Finally, because our data came just from Riyadh, we may not generalize our findings across Saudi Arabia. However, the current study is unique because it provides useful information about physical activity levels and factors associated with physical inactivity in a multi-ethnic community of young men living in Saudi Arabia.

## Conclusions

This study showed relatively high rates of physical inactivity among young men from Saudi Arabia and 11 Middle Eastern and Asian countries living in Saudi Arabia. The data revealed significant disparities in daily minutes spent on physical activities related to work, transport, recreation, vigorous and moderate-intensity physical activities and sedentary behaviors among study participants based on their nationalities. Participants' physical inactivity was significantly associated with nationality, age, residency period in Saudi Arabia, household type, education level, monthly income and BMI.

## Data Availability Statement

The raw data supporting the conclusions of this article will be made available by the authors, without undue reservation.

## Ethics Statement

The study protocol was approved by the Research Ethics Committee at Princess Nourah bint Abdulrahman University, Riyadh, Saudi Arabia. The patients/participants provided their written informed consent to participate in this study.

## Author Contributions

JA and NAlF: conceptualization. NAlK and JA: methodology. JA, NAlK, RA, and NAlF: software. JA and HA: validation. NAlF, NAls, and HA: formal analysis. RA: investigation and funding acquisition. NAlF: resources and supervision. NAlF and NAls: data curation. JA and RA: writing—original draft preparation. NAls and HA: writing—review and editing. JA: visualization and project administration. All authors have read and agreed to the published version of the manuscript.

## Funding

This research was supported by Princess Nourah bint Abdulrahman University Researchers Supporting Project number (PNURSP2022R34), Princess Nourah bint Abdulrahman University, Riyadh, Saudi Arabia.

## Conflict of Interest

The authors declare that the research was conducted in the absence of any commercial or financial relationships that could be construed as a potential conflict of interest.

## Publisher's Note

All claims expressed in this article are solely those of the authors and do not necessarily represent those of their affiliated organizations, or those of the publisher, the editors and the reviewers. Any product that may be evaluated in this article, or claim that may be made by its manufacturer, is not guaranteed or endorsed by the publisher.

## Abbreviations

BMI: body mass index
WHO: World Health Organization
GPAQ: Global Physical Activity Questionnaire
OR: odds ratio.
